# Effect of Heat Input on Microstructure Evolution and High-Cycle Fatigue Behavior of Ti-6Al-4V Fabricated by Wire-Laser Directed Energy Deposition

**DOI:** 10.3390/ma19183885

**Published:** 2026-09-11

**Authors:** Zhe Wang, Xi Chen, Meng Jiang, Zhijian Lu, Yuan Chen, Xuan Su, Qingfeng Yang, Dequan Yang, Peng He, Yanbin Chen

**Affiliations:** 1 State Key Laboratory of Precision Welding & Joining of Materials and Structures, Harbin Institute of Technology, Harbin 150001, Chinalzj2234616686@163.com (Z.L.); chenyuan@giim.ac.cn (Y.C.); chenyb@hit.edu.cn (Y.C.); 2 Zhengzhou Research Institute, Harbin Institute of Technology, Zhengzhou 450046, China; 3 Suzhou Research Institute, Harbin Institute of Technology, Suzhou 215128, China; 4 Maxphotonics Co., Ltd., Shenzhen 518125, China

**Keywords:** Ti-6Al-4V, wire-laser DED, heat input, α morphology, high-cycle fatigue, crack propagation

## Abstract

This work examines the response of Ti-6Al-4V fabricated by wire-laser directed energy deposition (DED) to heat inputs between 175 and 350 J/mm. Microstructure, tensile behavior, and rotating-bending fatigue were evaluated. As input increased, the transverse size of columnar prior β grains rose from about 642 to 1079 μm and the mean α-lath width increased from 0.65 to 1.32 μm. The α structure consequently changed from a fine acicular/basketweave form to a coarser lamellar form with clearer colonies. Tensile and yield strengths stayed near 900 and 840 MPa, whereas elongation fell from 14.3% to 10.3%. After a 600 °C, 4 h stress relief, fully reversed fatigue tests were performed at 500 MPa (R = −1). Specimens with 175 J/mm heat input reached about 3 × 10^7^ cycles, while one with 350 J/mm fractured at roughly 1 × 10^5^ cycles. The longer life of H175 coincided with its finer interwoven α structure, closer striations, more secondary cracks, and a more tortuous crack route.

## 1. Introduction

Aerospace structures commonly use Ti-6Al-4V because the alloy combines low density with corrosion resistance and useful mechanical strength. Blisks can nevertheless accumulate wear, impact damage, tip loss, and fatigue damage during thermomechanical service. Repairing the affected area by additive manufacturing can save material and avoid replacing an entire component [1,2,3,4]. For directed energy deposition (DED)-repaired blisks, however, structural acceptance is complicated by process defects, residual stress, anisotropic structure, and locally variable fatigue sensitivity [5,6,7,8]. A wire-laser DED process is attractive for such repairs because material can be deposited into a localized region with efficient feedstock use and flexible path control [9,10,11,12]. Wire-laser DED can produce dense Ti-6Al-4V parts with a continuous microstructural transition between the deposited material and the substrate/heat-affected zone [11,12,13]. Meanwhile, the laser-based process concentrates heat in a smaller molten pool, which aids geometrically controlled repair. The final structure and properties remain strongly dependent on the deposition thermal cycle because titanium alloys respond sensitively to temperature history [14,15,16].

Deposition heat input governs thermal accumulation, cooling rate, solidification, and phase transformation in additively manufactured Ti-6Al-4V. Successive deposition layers impose repeated thermal cycles that can alter prior β grain morphology, α-phase characteristics, layer-band features, and local mechanical behavior [17,18,19,20,21,22,23]. Previous work has shown that thermal accumulation and interlayer cooling time can markedly affect the temperature field, cooling rate, and forming quality in GMAW-based additive manufacturing [17] and has related DED anisotropy to directional solidification and cyclic reheating [4]. In wire-based processing, interpass cooling has been shown to affect both microstructure and tensile behavior [24], while heat input and laser power can modify prior β grain morphology and the mechanical response [25,26,27]. Thus, the thermal history generated by the deposition process is central to the final microstructural state.

The arrangement of the α phase has a direct bearing on deformation and fatigue-crack resistance in Ti-6Al-4V. Fine acicular α or martensitic α′ creates numerous α/β interfaces that can deflect an advancing crack, whereas coarse lamellar colonies offer more continuous routes for localized slip and crack extension [28,29,30,31]. Earlier studies have related microstructural refinement to higher tensile and fatigue strength [28], in line with the scale effect described by the Hall–Petch relationship [30]. Heat-input-driven changes in prior β grains and α-phase configuration should therefore influence the mechanical reliability of wire-laser DED deposits.

For blisk applications, cyclic durability, especially high-cycle fatigue, is a central requirement [32]. Defect population, residual stress, prior β grain size, α morphology, crystallographic variation, and crack-route geometry act together to determine fatigue behavior in additively manufactured Ti-6Al-4V [33,34,35,36,37,38,39,40]. Studies of wire-laser DED deposits have linked fatigue fracture to metallurgical and fractographic features [35], whereas wire-laser DED material may show coupled effects of microstructure and defects [36]. In selective laser melting, both low- and high-cycle responses are also sensitive to defect state and mean stress [34,38]. The effect of heat input on the prior β grain/α morphology combination and rotating-bending fatigue of wire-laser DED Ti-6Al-4V, however, remains to be clarified.

The experimental program was established to determine how nominal heat input influences structure and fatigue in wire-laser DED Ti-6Al-4V. Four input levels were obtained through different travel speeds, with laser power and wire-feed speed unchanged. Prior β grain characteristics, α-phase features, tensile properties, and fracture appearances were measured first. The two most distinct material states were subsequently assessed by fully reversed rotating-bending fatigue tests (R = −1), followed by fracture-surface and crack-growth examination. The analysis focuses on the link between heat-input-induced structural changes and life at a fixed stress level.

## 2. Materials and Methods

Commercial Ti-6Al-4V (substrate: Baoji Titanium Industry Co., Ltd., Baoji, China; feedstock wire: Institute of Metal Research, Chinese Academy of Sciences, Shenyang, China) was used for both the substrate and the feedstock wire. The substrate measured 200 mm × 100 mm × 10 mm. Before deposition, its surface was mechanically cleaned to remove contamination, moisture, and oxide films and was subsequently wiped with ethanol (Shanghai Aladdin Biochemical Technology Co., Ltd., Shanghai, China). The filler wire was 1.0 mm in diameter. The chemical compositions of the substrate and wire are provided in Table 1.

**Figure 1:**
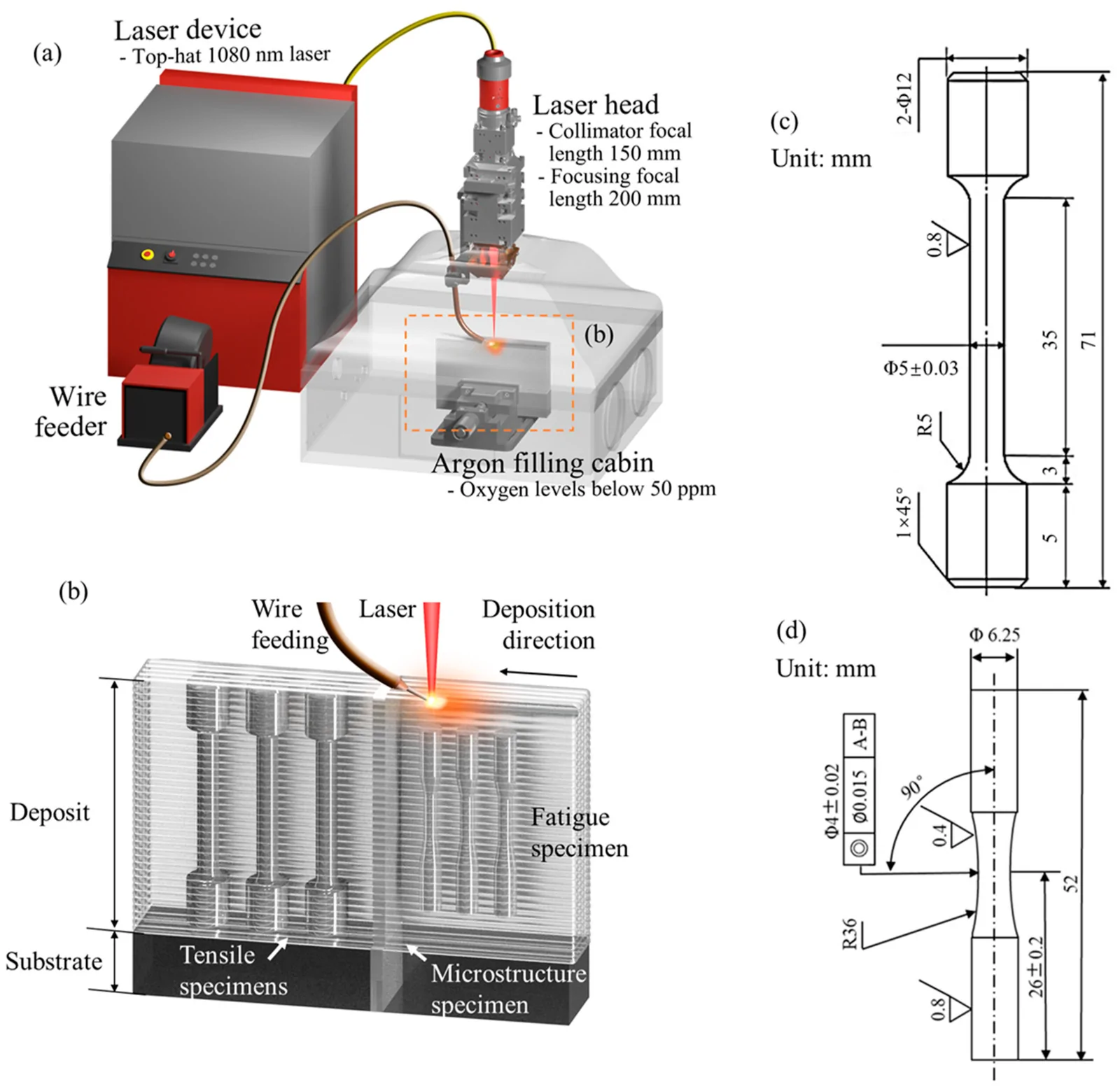
Schematic of the wire-laser DED system and specimen extraction. (**a**) Wire-laser DED setup; (**b**) locations of microstructure and mechanical properties specimens; (**c**) geometry of tensile specimen; and (**d**) geometry of rotating bending fatigue specimen.

Deposits were manufactured with the wire-laser DED system illustrated in Figure 1. The apparatus comprised a Laserline LDF diode laser (Laserline GmbH, Mülheim-Kärlich, Germany) with a maximum output of 6 kW, a 1080 nm wavelength top-hat energy profile, a Laserline OTS-5 processing head (Laserline GmbH, Mülheim-Kärlich, Germany) with a 200 mm focusing focal length, a wire-feeding unit (KD 7000, Fronius International GmbH, Wels, Austria), a six-axis robot (KUKA KR 60, KUKA Roboter GmbH, Augsburg, Germany), and a custom-built argon-filled enclosure. All experiments were conducted at a defocus distance of +10 mm, with the focal plane located 10 mm above the working plane. Under this condition, the beam diameter was 1.76 mm, corresponding to an irradiated area of 2.43 mm^2^. With the laser power fixed at 1400 W, the nominal power density was 0.58 kW/mm^2^. During deposition, the laser head and wire-delivery nozzle moved synchronously. All builds were conducted in an argon-filled enclosure as Ti-6Al-4V is readily oxidized at elevated temperatures. Argon (99.9% purity; Harbin Liming Gas Co., Ltd., Harbin, China) was supplied continuously during deposition to maintain a positive-pressure atmosphere. The oxygen level in the enclosure was maintained below 50 ppm, as monitored using a Microx oxygen analyzer (Ntron Gas Measurement Ltd., Navan, Ireland).

Four deposition settings were used to generate different thermal histories in Ti-6Al-4V. Only travel speed was adjusted; laser power, wire-feed rate, defocus, deposition path, and shielding were fixed. The laser power and wire-feed speed remain unchanged as 1400 W and 70 cm/min, respectively. The nominal input was calculated as the laser energy delivered per unit travel distance:

*E* = *P*/*v* (1)

where *E* denotes nominal heat input, *P* is laser power, and *v* is deposition speed. Unless otherwise specified, the term heat input in the following sections refers to this nominal parameter. The sample labels H175, H233, H290, and H350 correspond to nominal heat inputs of 175, 233, 290, and 350 J/mm, respectively, as shown in Table 2. As the laser power and beam diameter were unchanged, the nominal heat input was varied solely through the deposition speed. To make this effect explicit, the nominal laser-spot irradiation time at a fixed material position was calculated as

*t_i_* = *d*/*v* (2)

where *d* is the 1.76 mm beam diameter. The irradiation times for H175 and H350 were 0.22 s and 0.44 s, respectively. Thus, a lower deposition speed extends the time for which a local material position is exposed to the laser spot. This increases the local energy delivered during an individual pass and consequently increases the nominal heat input. The resulting difference in local thermal exposure provides the basis for the heat-input-dependent microstructural evolution discussed below.

Metallographic coupons were removed from the Ti-6Al-4V builds by wire electrical-discharge machining (DK7740C-CT, Suzhou Baoma Numerical Control Equipment Co., Ltd., Suzhou, China). Preparation comprised sequential SiC grinding from 80# to 2000# (VSM · Vereinigte Schmirgel- und Maschinen-Fabriken AG, Hanover, Germany), polishing with 2.5 μm diamond suspension (Zhejiang Lixie Instrument Equipment Co., Ltd., Yiwu, China) to a mirror finish, and Kroll etching (using reagents supplied by Shanghai Aladdin Biochemical Technology Co., Ltd., Shanghai, China) for approximately 30 s. A ZEISS Stemi 508 optical microscope (Carl Zeiss Microscopy GmbH, Jena, Germany) was used for macrostructural observations. Prior β grains and α-phase configurations were examined by SEM (SU5000, Hitachi High-Tech Corporation, Tokyo, Japan), and dimensional and morphological measurements were taken from the optical and electron micrographs.

Tensile specimens were machined parallel to the build direction. Three specimens were tested for each heat-input condition. Tests were performed at room temperature on a universal testing machine (Instron 5569, Instron, Norwood, MA, USA) with a crosshead speed of 0.375 mm/min. Tensile tests were conducted in accordance with GB/T 228.1-2021. Ultimate tensile strength (UTS), yield strength (YS), and elongation (EL) were recorded for comparison.

The fatigue comparison used H175 and H350, which represented the two most different microstructural states. Before specimen machining, both deposits underwent the same 600 °C stress-relief treatment for 4 h in a KSL-1000X-M furnace (Hefei Kejing Materials Technology Co., Ltd., Hefei, China) and were furnace cooled. This procedure limited the influence of residual stress and placed the two conditions on a common post-deposition basis. Fine α-related details can change during subtransus exposure, but the established prior β grain framework is retained. Post-fatigue sections continued to show a clear α-morphology difference between H175 and H350. Three round bars were tested per condition, and the fatigue lives obtained for the replicates were of the same order of magnitude. Room-temperature rotating-bending fatigue tests were conducted in accordance with GB/T 4337-2015 using a PQ-60 machine (Jinan MTS Test Technology Co., Ltd., Jinan, China) at a stress amplitude of 500 MPa with R = −1, and complete separation was recorded as failure. The fixed stress amplitude was selected to compare the fatigue response of deposits with different heat-input-induced microstructures. Similar 500 MPa stress-amplitude conditions have been used in fatigue studies of additively manufactured Ti-6Al-4V [41,42].

Following tensile and fatigue testing, fracture surfaces were characterized by SEM. Tensile fractography was used to identify fracture mode and differences in plastic deformation. For fatigue specimens, the initiation, propagation, and final-fracture regions were examined. Cross-sectional observations adjacent to fatigue fracture surfaces were additionally used to relate α morphology to crack-propagation behavior.

## 3. Results

### 3.1. Microstructural Characteristics

**Figure 2:**
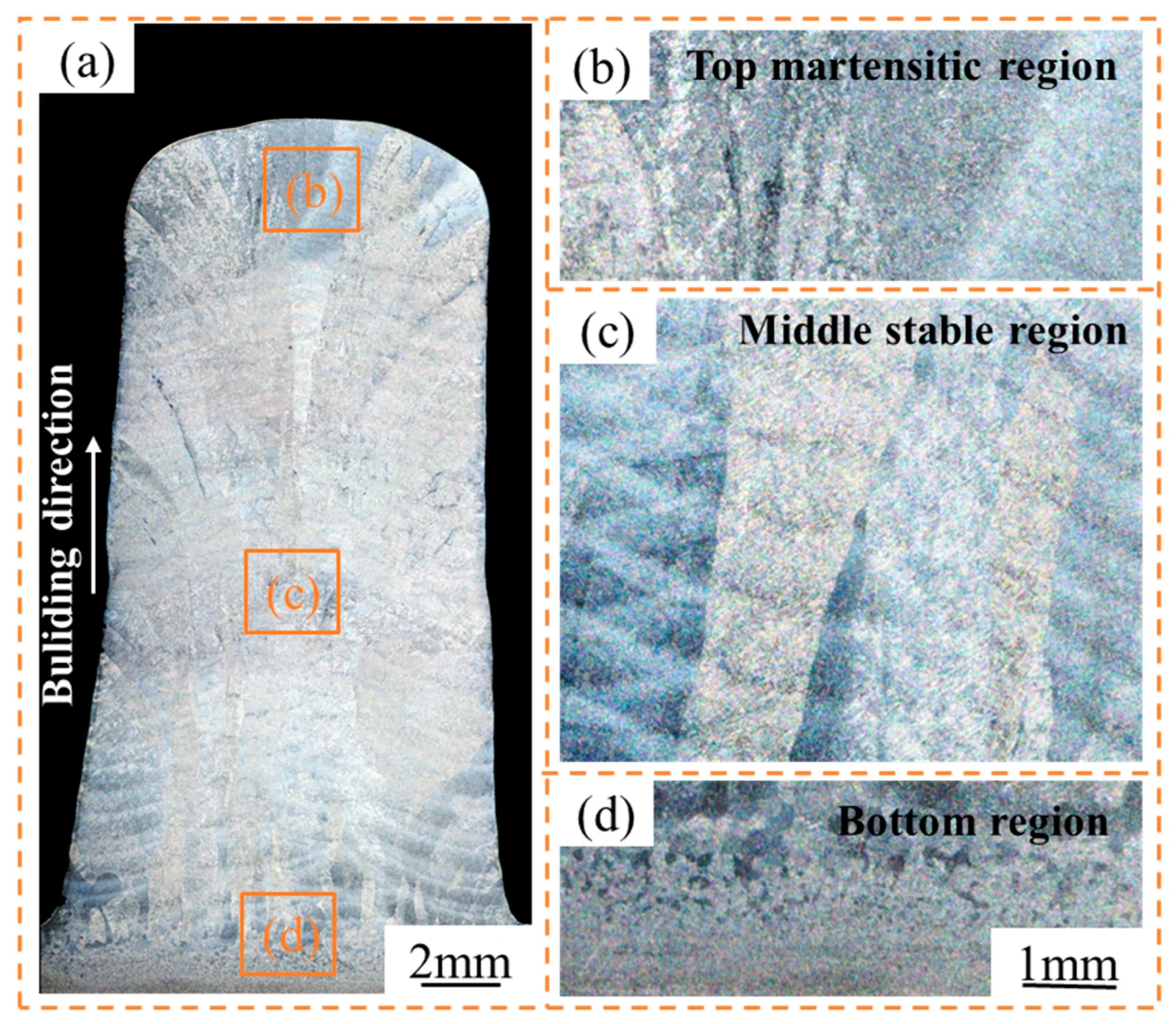
Macrostructure of the wire-laser DED Ti-6Al-4V deposit. (**a**) Overall cross-sectional morphology; (**b**) top martensitic region; (**c**) middle stable region; and (**d**) bottom region.

**Figure 3:**
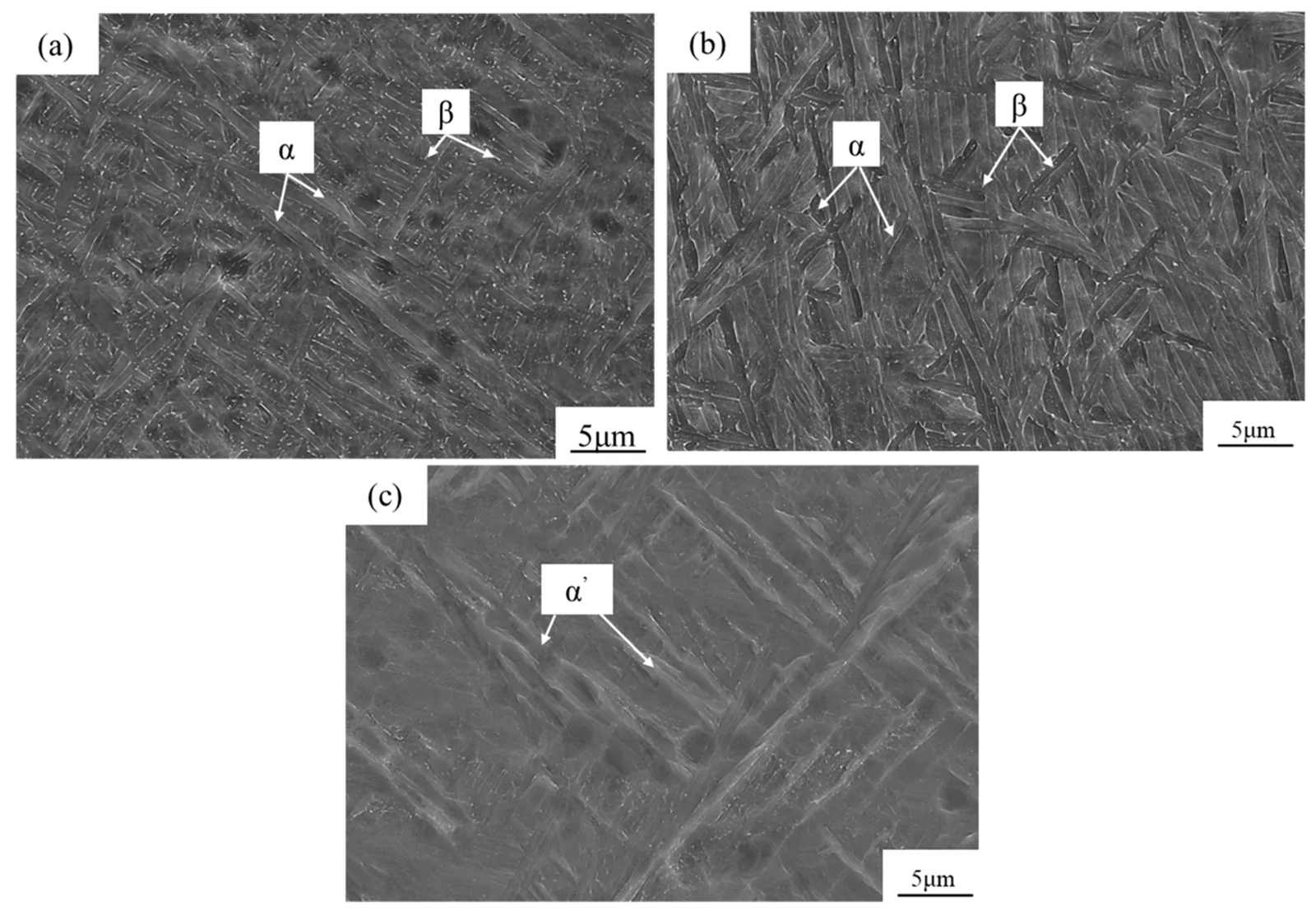
Microstructures in different regions of the wire-laser DED Ti-6Al-4V deposit. (**a**) Bottom region; (**b**) middle stable region; and (**c**) top martensitic region.

A clear through-height structural variation was present in the deposited wall (Figure 2). The lower portion contained fine equiaxed prior β grains, the central portion showed stable columnar prior β growth, and the upper portion was martensitic. Figure 3 reveals distinct microstructures in the three deposition regions. The fine-grained bottom region contained relatively fine and irregular α within the prior β grains. In the middle stable region, acicular α was distributed throughout columnar prior β grains. The top region contained abundant acicular martensitic α′, consistent with rapid cooling near the end of deposition. The spatial variation in α/α′ morphology reflects the different thermal histories experienced along the build height. Representative metallographic cross-sections in the present study showed no macroscopically observable pores or lack-of-fusion defects. A continuous microstructural transition was observed between the deposited material and the substrate/heat-affected zone. With specimens mainly extracted from the middle stable region, this work treats the continuous transition as a prerequisite and focuses on the heat-input-dependent microstructure and mechanical properties of the deposited material. The following microstructural discussion emphasizes the α-phase features of that stable region.

**Figure 4:**
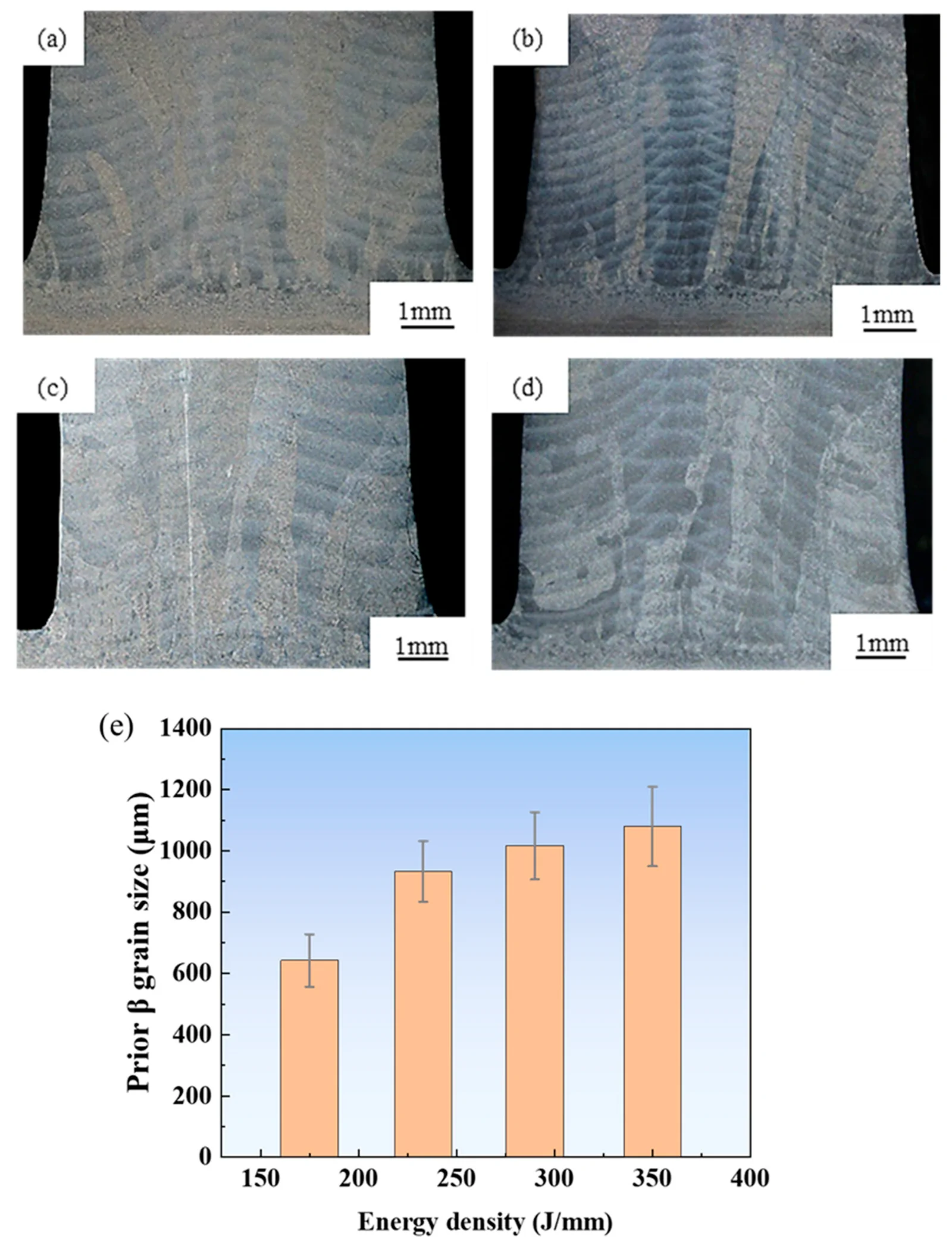
Effect of heat input on prior β grain morphology of wire-laser DED Ti-6Al-4V deposits. (**a**) 175 J/mm; (**b**) 233 J/mm; (**c**) 290 J/mm; (**d**) 350 J/mm; and (**e**) average prior β grain width.

Figure 4 summarizes the dependence of prior β grain morphology on heat input. All stable regions retained columnar prior β grains, but their transverse width increased as heat input rose. The mean width increased from about 642 μm at 175 J/mm to about 1079 μm at 350 J/mm. Although this trend demonstrates heat-input-induced coarsening of prior β grains, the intragranular α morphology is the microstructural variable more directly linked to the mechanical responses discussed below.

**Figure 5:**
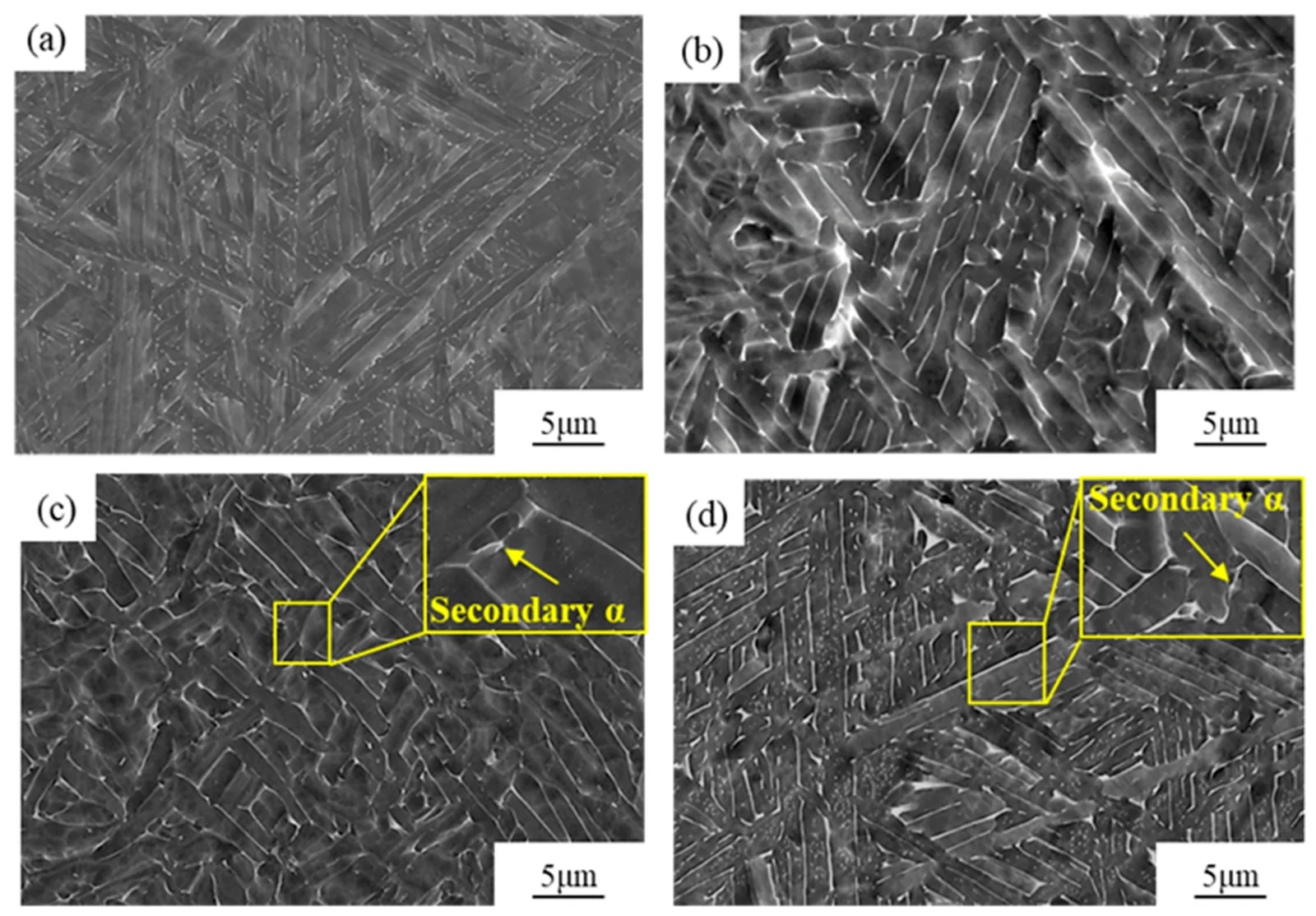
Stable-region α-phase configurations in wire-laser DED Ti-6Al-4V produced at nominal inputs of (**a**) 175 J/mm, (**b**) 233 J/mm, (**c**) 290 J/mm, and (**d**) 350 J/mm.

Figure 5 shows the α-phase state in the stable region for all four input levels. Basketweave α with retained β between lamellae was present in every condition, but its scale and connectivity changed appreciably. At lower input, the α network was compact and interwoven; higher input generated a coarser intragranular lamellar structure. Fine acicular α/α′ dominated H175, while H233 remained largely acicular/basketweave but slightly coarser. H290 and H350 displayed a stronger lamellar character, clearer α colonies, and more secondary α. These observations demonstrate the pronounced influence of nominal heat input on α morphology in this deposition route.

**Figure 6:**
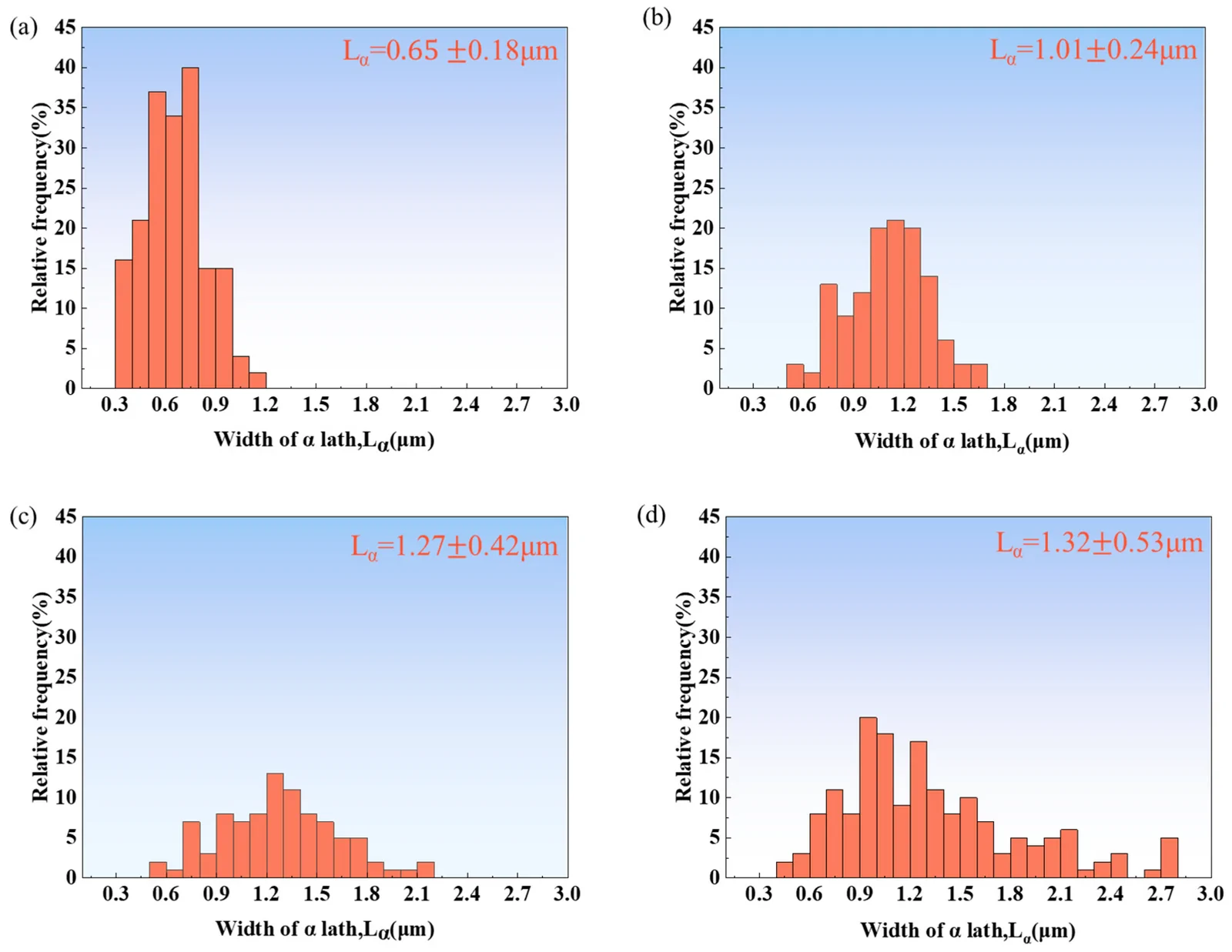
Quantitative variation in α lath width as a function of heat input. (**a**) 175 J/mm; (**b**) 233 J/mm; (**c**) 290 J/mm; and (**d**) 350 J/mm.

The α-lath measurements in Figure 6 corroborate this coarsening trend. Mean lath width increased from approximately 0.65 μm at 175 J/mm to 1.32 μm at 350 J/mm, confirming that greater heat input coarsened the intragranular α phase. The change was not limited to lath size: the prevailing morphology progressed from fine acicular α/α′ at low heat input to coarser lamellar α with more apparent α colonies and secondary α at high heat input. Accordingly, α-phase evolution, rather than prior β grain coarsening alone, is considered the principal microstructural change relevant to subsequent tensile and fatigue behavior.

### 3.2. Tensile Properties and Fracture Behavior

**Figure 7:**
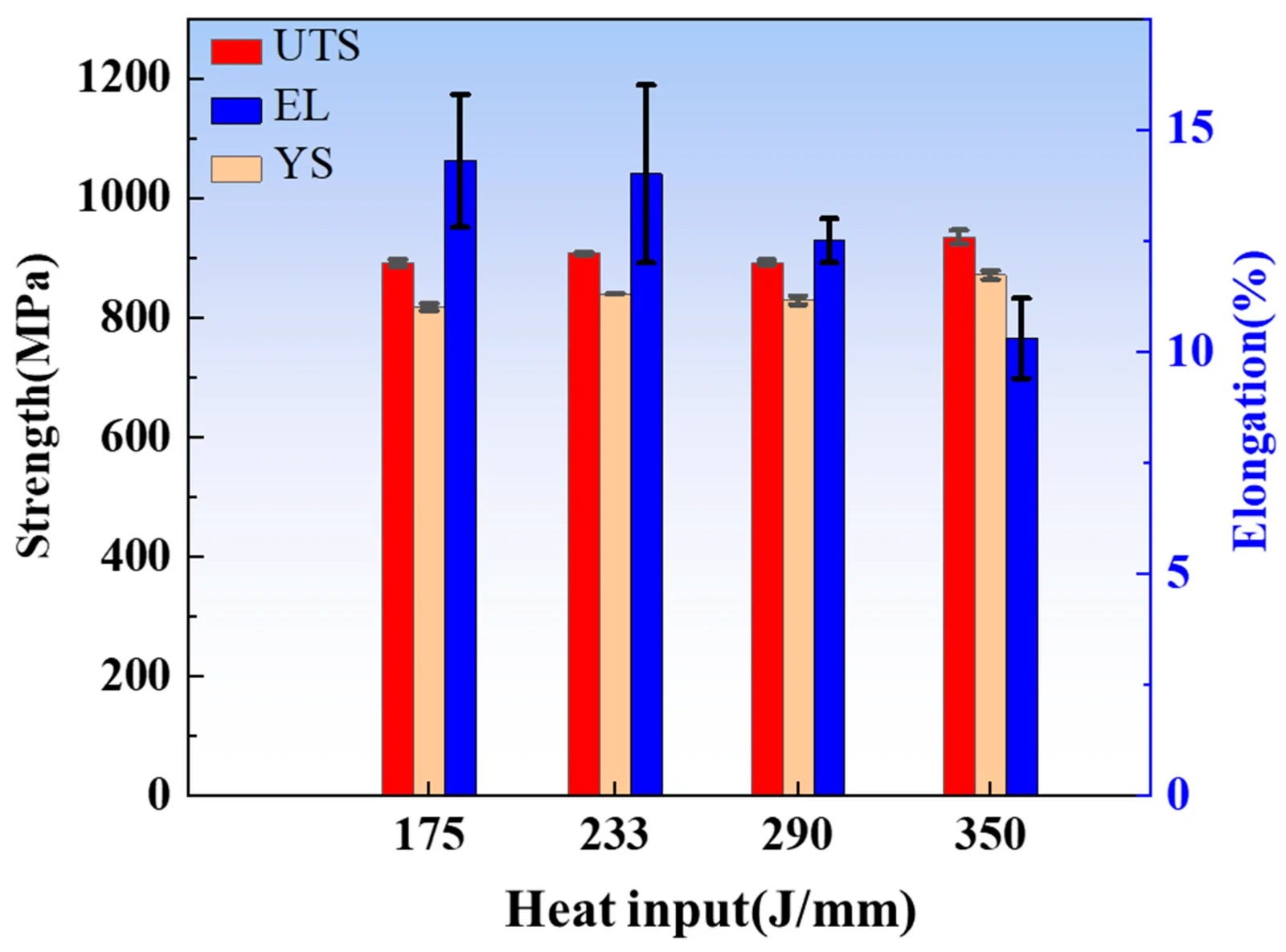
Tensile properties of wire-laser DED Ti-6Al-4V deposits fabricated under different heat inputs.

The tensile data in Figure 7 show that strength was comparatively insensitive to the investigated heat-input range. Ultimate tensile strength remained near 900 MPa and yield strength near 840 MPa, with no monotonic heat-input dependence. Ductility, in contrast, decreased progressively: elongation was 14.3% and 14.0% for H175 and H233, respectively, but decreased to 12.5% for H290 and 10.3% for H350. Thus, the elongation of H350 was approximately 28% lower than that of H175, indicating that higher heat input primarily reduced ductility rather than strength.

**Figure 8:**
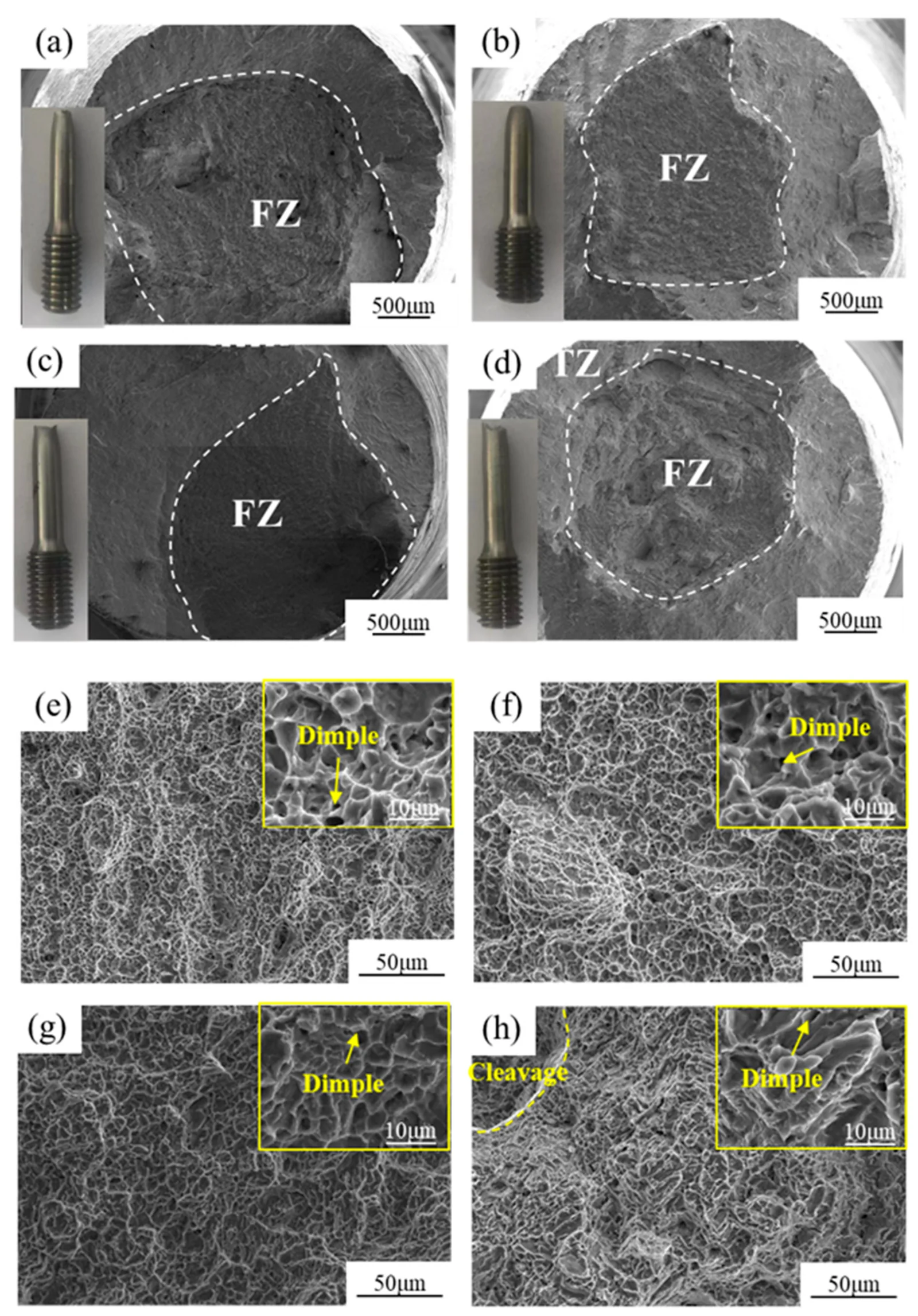
Tensile fracture morphologies of wire-laser DED Ti-6Al-4V deposits under different heat inputs. (**a**–**d**) Macroscopic fracture appearances of H175, H233, H290 and H350 samples; (**e**–**h**) SEM fracture morphologies of H175, H233, H290 and H350 samples.

The macroscopic fracture surfaces in Figure 8 comprised a central fibrous zone (FZ) surrounded by a tearing zone (TZ). H175 and H233 showed pronounced necking and a larger FZ, consistent with their greater elongation. Necking was less apparent in H290 and H350, which also displayed reduced macroscopic plastic deformation. As heat input rose, the FZ occupied a smaller fraction of the surface and the TZ became more prominent, matching the measured ductility loss.

SEM examination showed dimpled, ductile fracture in all four conditions, although the dimple characteristics changed with heat input. H175 and H233 contained many fine, deep, nearly equiaxed dimples, consistent with their larger plastic strain before rupture. In contrast, H290 and H350 exhibited coarser, shallower shear dimples; isolated cleavage facets were also observed in H350. These features agree with the minimum elongation measured for H350.

Across the tensile data and fracture observations, ductility was the property most affected by heat input, whereas strength changed little. Lower-input material combined fine α features with more elongation and stronger necking. The coarse lamellar α state at high input was accompanied by reduced elongation and shallower dimples. These differences provide the mechanical context for the fatigue comparison, in which crack nucleation and propagation are additionally important.

### 3.3. High-Cycle Fatigue Response and Crack Propagation Characteristics

All specimens failed during fully reversed rotating bending at 500 MPa and R = −1. The H175 condition lasted approximately 3 × 10^7^ cycles; H350 failed near 1 × 10^5^ cycles. Thus, after the same stress-relief schedule, H175 exhibited a life more than two orders of magnitude longer. Since only one stress level was used, the dataset compares durability at 500 MPa and does not establish an S–N curve or an endurance limit.

**Figure 9:**
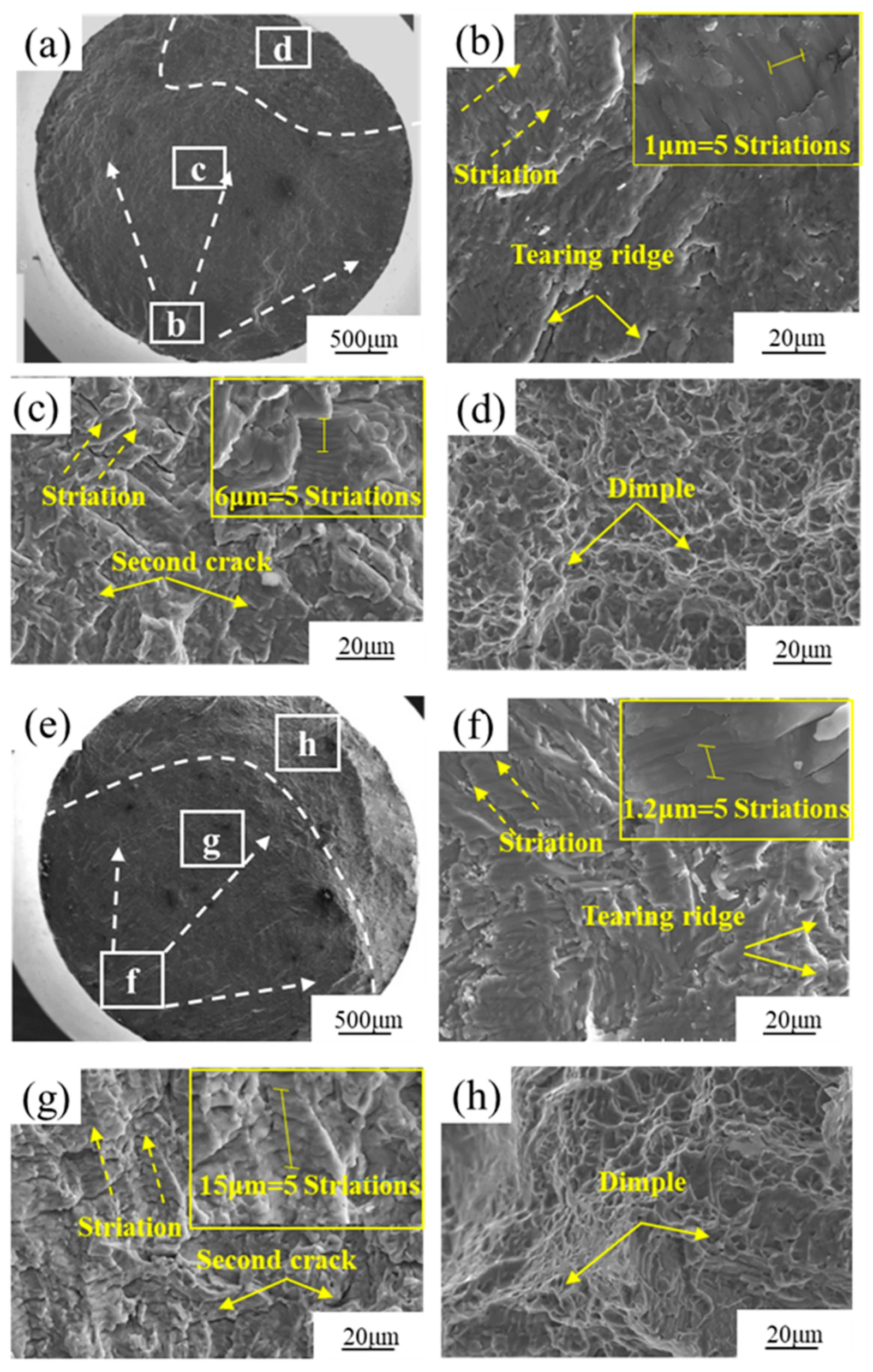
Fatigue fracture morphologies of wire-laser DED Ti-6Al-4V deposits after high-cycle fatigue testing under rotating-bending loading at a stress amplitude of 500 MPa and R = −1. (**a**–**d**) H175 sample: overall fracture surface, near crack initiation region, crack propagation region and final fracture region; (**e**–**h**) H350 sample: overall fracture surface, near crack initiation region, crack propagation region and final fracture region.

Figure 9 shows the fatigue fracture surfaces after high-cycle testing in the rotating-bending mode. Both materials displayed identifiable initiation, propagation, and final-fracture regions. In H175, fine striations and tearing ridges were evident near initiation, where the striation spacing was approximately 0.2 μm. The propagation region contained coarser striations with a spacing of approximately 1.2 μm together with secondary cracks. The final-fracture region was dominated by dimples, indicating rapid overload after fatigue-crack growth.

For H350, striations were farther apart than in H175 near both the initiation and propagation zones, at roughly 0.24 μm and 3 μm, respectively. Propagating cracks in H350 also generated fewer secondary branches. These features are consistent with faster local advance and the shorter observed life. The fracture surface additionally contained a larger final-overload area and a smaller stable-propagation area, supporting the conclusion that the stable-growth period was reduced.

**Figure 10:**
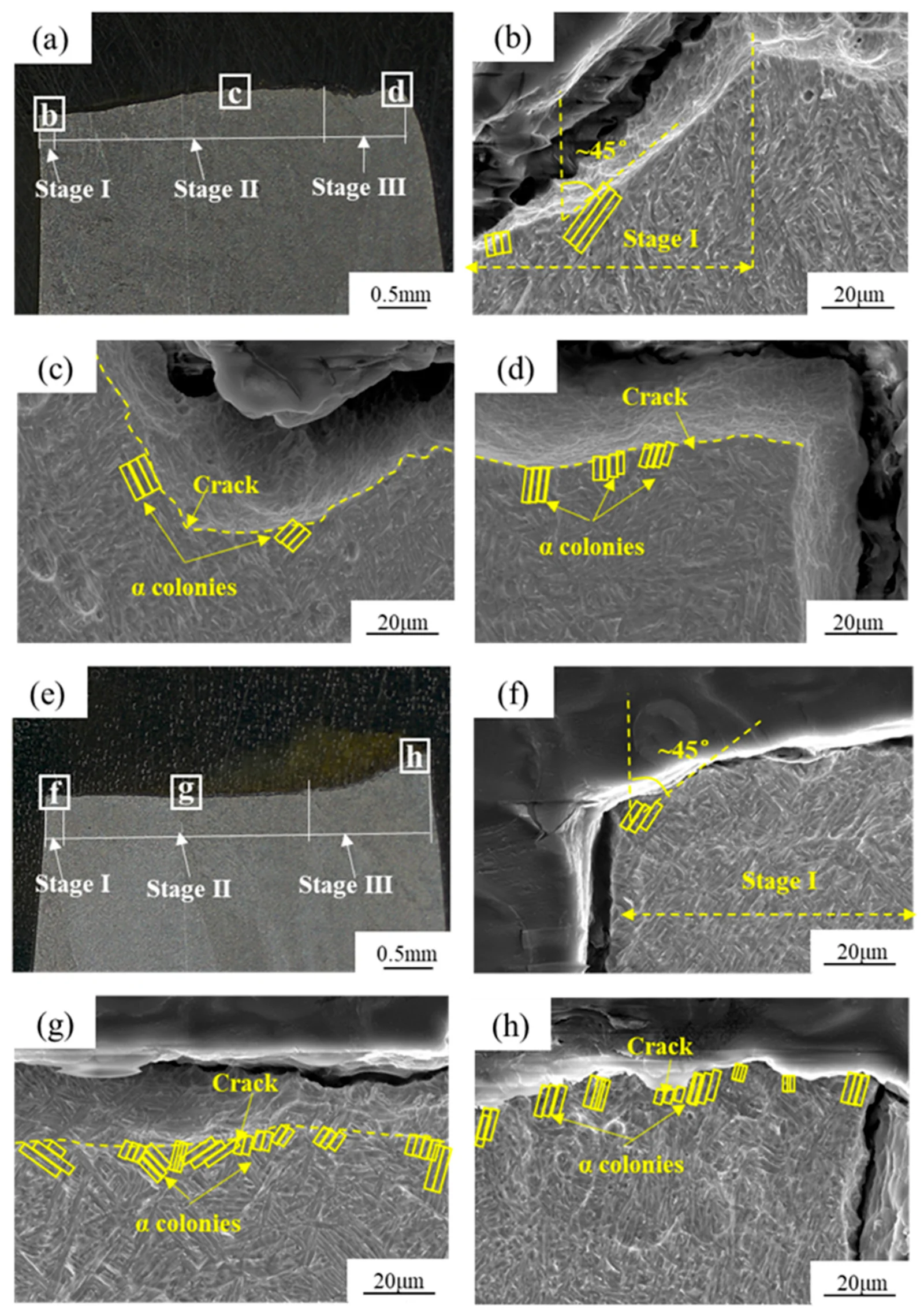
Fatigue crack propagation characteristics near the fracture surface of wire-laser DED Ti-6Al-4V deposits. (**a**–**d**) H175 sample: macroscopic crack path, crack initiation region, crack propagation region and final fracture region; (**e**–**h**) H350 sample: macroscopic crack path, crack initiation region, crack propagation region and final fracture region.

Crack paths close to the fatigue surfaces are presented in Figure 10. In H175, surface-near initiation occurred where α colonies had local alignment. The early crack followed colony boundaries at an angle of about 45° to the loading direction, then followed an increasingly tortuous course. Basketweave α occupied most of the adjacent material, and colonies were limited near the crack boundary. The irregular route and secondary cracking show that local α morphology repeatedly impeded propagation.

H350 also initiated fatigue cracks close to the surface in locally aligned α-colony areas. Relative to H175, colonies were more evident in both the propagation and final-fracture zones. The crack followed the lamellar-colony arrangement with less deviation, and its propagation surface was smoother. This microstructure therefore offered more continuous local channels for subsequent growth and terminal fracture.

**Figure 11:**
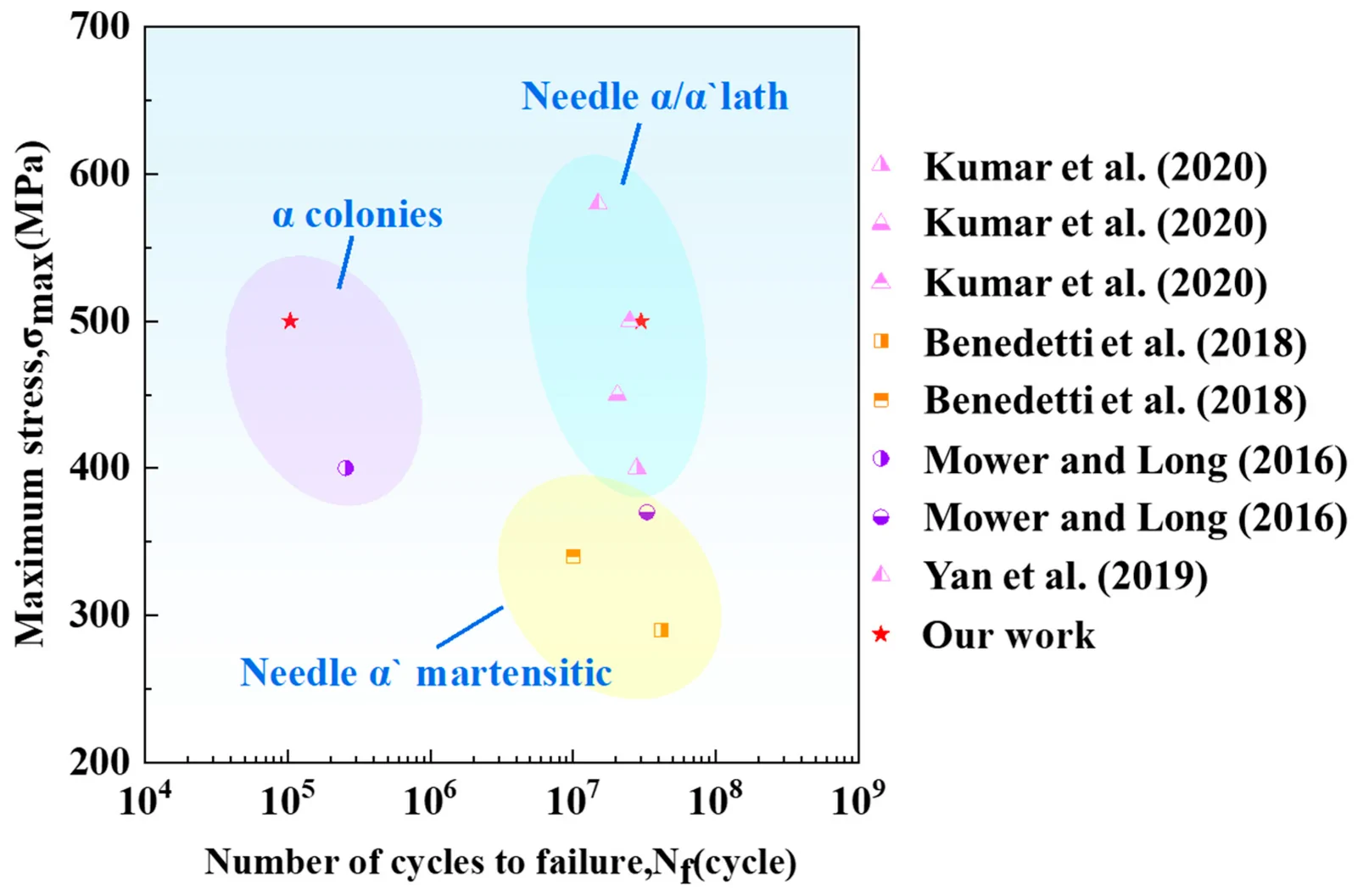
Present high-cycle fatigue results in relation to literature data for additively manufactured Ti-6Al-4V with different microstructural morphologies [34,38,39,40].

Figure 11 positions the 500 MPa data from this work against published high-cycle fatigue results for additively manufactured Ti-6Al-4V with different microstructural states [34,38,39,40]. The fine acicular/basketweave α structure of H175 supported about 3 × 10^7^ cycles and compared well with the literature dataset. At the same applied amplitude, the coarse, colony-rich H350 state fractured at around 1 × 10^5^ cycles. The comparison indicates that α configuration and heat-input selection are relevant to fatigue resistance in wire-laser DED.

At the selected high-cycle fatigue condition, H175 consistently outperformed H350. Its fracture surfaces showed narrower local striations, more secondary cracks, and a larger propagation region; H350 showed the opposite combination of wider striations, fewer secondary cracks, and a larger final-fracture region. Together with the observed crack paths, these results associate the life difference with the retained morphology change from fine acicular/basketweave α in H175 to coarser lamellar α with developed colonies in H350.

## 4. Discussion

### 4.1. Heat-Input-Controlled α Morphology Evolution

The results demonstrate that heat input substantially alters the microstructure of wire-laser DED Ti-6Al-4V. Increasing the heat input widened prior β grains, evidencing a modified solidification and thermal history. More importantly, α morphology changed from fine acicular/basketweave α at 175 J/mm to coarse lamellar α at 350 J/mm, together with clearer α colonies and secondary α. Because α morphology affects both deformation and fatigue-crack propagation, the thermal origins of these α-related changes are considered below.

**Figure 12:**
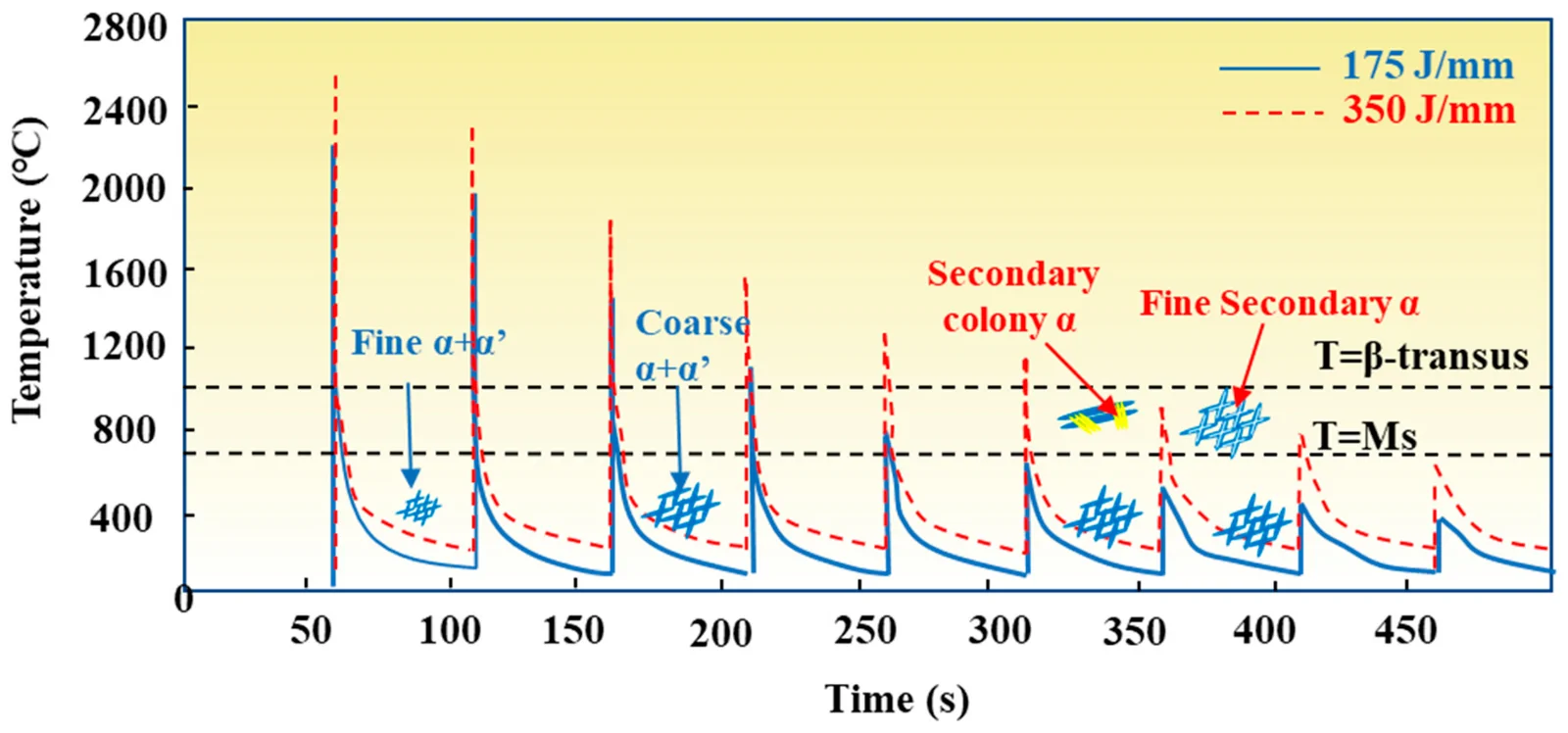
Qualitative temperature-time schematic for a representative point in the stable deposited region during multi-layer and multi-track wire-laser DED.

During multi-layer and multi-track wire-laser DED, a qualitative temperature-time schematic for a representative point in the stable region is illustrated in Figure 12. It shows the successive heating and cooling cycles caused by initial deposition and reheating from subsequent passes. The schematic visualizes how nominal heat input changes the duration of high-temperature exposure and the extent of subsequent reheating. These repeated cycles provide an in situ heat-treatment effect that influences α/β transformation and α-phase evolution, thereby linking nominal heat input to the final microstructure and mechanical response. The profiles are intended to qualitatively compare the relative thermal exposure associated with different nominal heat inputs rather than to reproduce direct temperature measurements. A lower input gives a shorter high-temperature exposure and more rapid cooling after melting, conditions that promote fine acicular α/α′ and compact basketweave α. A higher input increases heat accumulation and repeated reheating by subsequent layers, extending the period available for α/β evolution. The resulting longer exposure favors coarser α and pronounced lamellae, while reheating assists secondary-α precipitation and colony development. This framework is consistent with the observations for H290 and H350.

**Figure 13:**
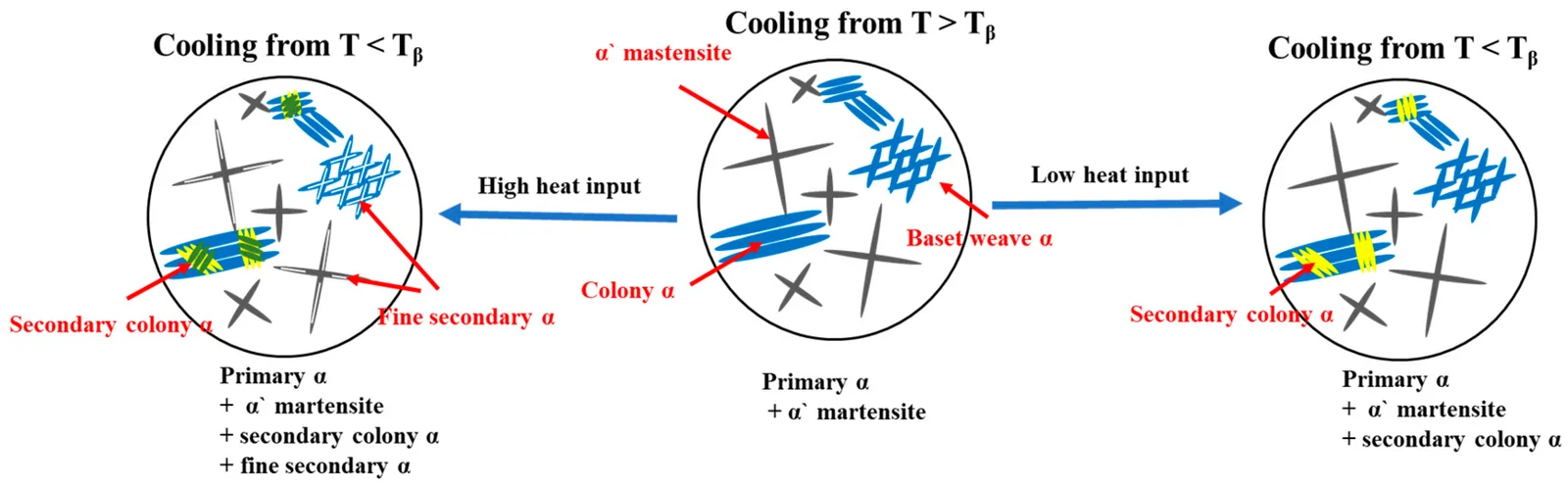
Schematic illustration of α phase evolution during repeated thermal cycling in wire-laser DED Ti-6Al-4V deposits.

Figure 13 summarizes the evolution of α-related constituents during repeated thermal cycling. On cooling from above the β-transus, prior β transforms to primary α and martensitic α′. Reheating during subsequent layers can decompose part of the metastable α′ and allow retained β to contribute to secondary-α precipitation. Repetition of these cycles produces a mixed structure comprising primary α, residual or decomposed α′, secondary α, and α colonies. The final morphology is therefore governed by both initial cooling after solidification and the later reheating history.

This thermal-cycle framework explains the contrasting structures at 175 and 350 J/mm. Low heat input favored a fine acicular/basketweave α network, whereas high heat input promoted coarser lamellar α, larger colonies, and secondary α. Heat input thus changed not only α-lath width but also the arrangement and continuity of the α phase. This distinction is consequential because Ti-6Al-4V responds to the scale and spatial organization of α. The low-heat-input material retained a refined, interwoven framework; the high-heat-input material contained coarser lamellae and more continuous colonies. The heat-input effect should therefore be understood as a combined change in α fineness, lamellar continuity, colony development, and secondary-α distribution.

**Figure 14:**
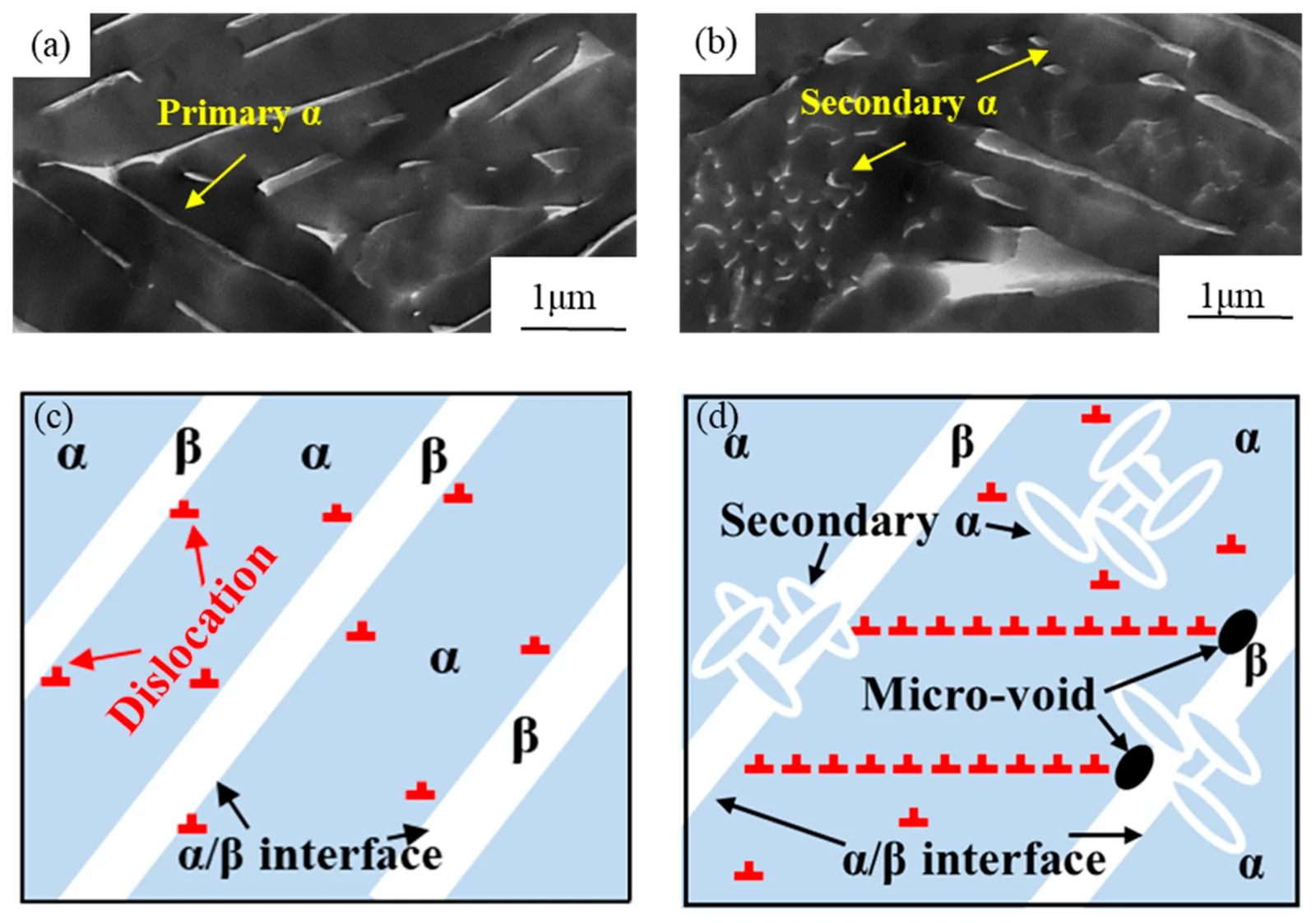
SEM evidence of tensile fracture and conceptual damage diagrams for wire-laser DED Ti-6Al-4V: (**a**,**c**) H175; (**b**,**d**) H350.

The interpretation in Figure 14 links tensile fracture behavior to the α arrangement. Fine acicular/basketweave α and frequent α/β interfaces in H175 favor a more distributed plastic response, consistent with higher elongation and deeper dimples. In H350, coarse lamellae and developed colonies promote localized deformation and microvoid formation, in agreement with lower elongation and shallower dimples. This mechanism is compatible with the fracture observations in Figure 8.

Nominal heat input altered α morphology by changing cooling rate, heat accumulation, and the extent of repeated reheating. Lower input retained a fine acicular/basketweave network. Higher input produced coarser lamellae, more visible colonies, and additional secondary α. The resulting structural transition accounts for the loss of tensile ductility and contributes to the large fatigue-life difference between H175 and H350.

Changing travel speed at fixed wire-feed rate altered nominal heat input, the wire-feed/travel-speed ratio, and the amount of feedstock supplied per deposited length. These coupled changes could affect bead geometry and heat storage. Each parameter set nevertheless yielded a stable build, and all specimens were sampled from the same stable region. The measured structural differences are therefore discussed as consequences of the overall thermal history, not as an effect of laser energy in isolation.

### 4.2. Heat-Input-Controlled Fatigue Crack Propagation Behavior

H175 and H350 showed strongly different lifetimes under the 500 MPa rotating-bending condition. Both groups received identical stress relief before machining. Although such treatment can modify fine α features, Figure 10 confirms that the dominant contrast persisted: H175 was finer and more interwoven, whereas H350 remained coarse, lamellar, and colony rich. Fatigue behavior is therefore considered in relation to these retained morphology differences.

**Figure 15:**
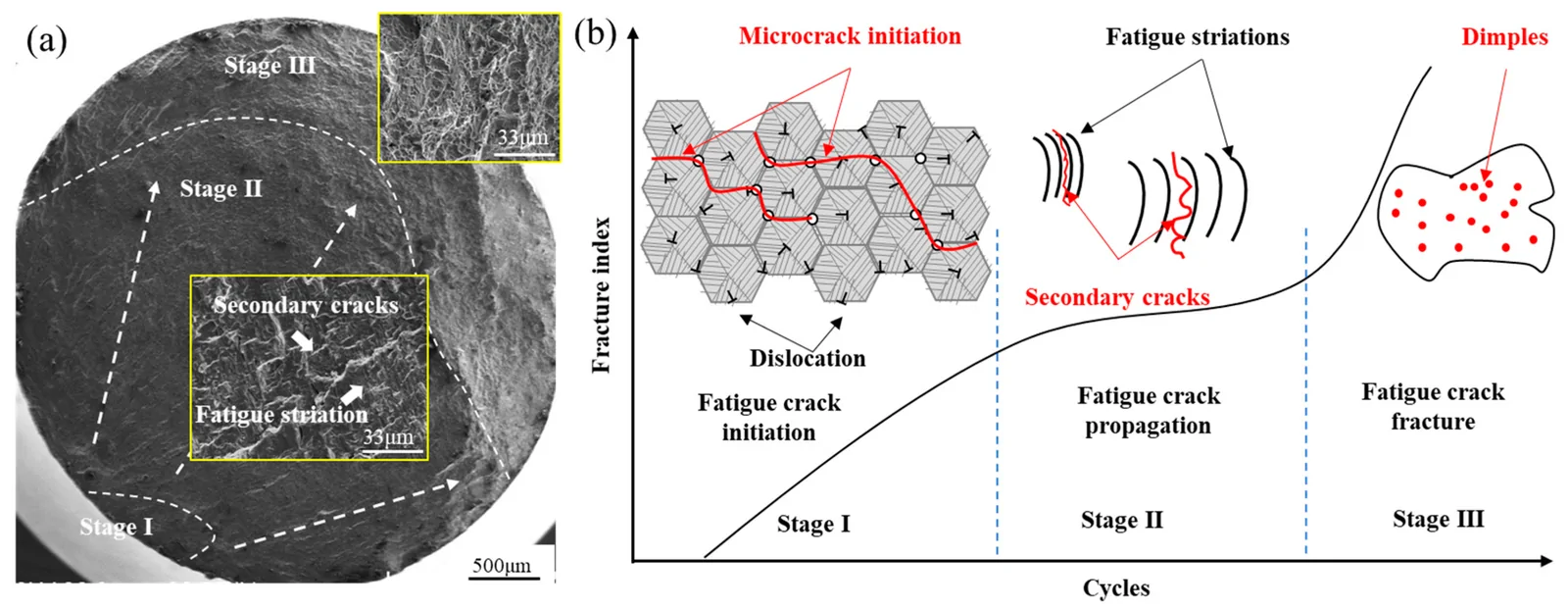
Conceptual sequence of rotating-bending fatigue failure in wire-laser DED Ti-6Al-4V: (**a**) fracture-surface zones; (**b**) crack-growth route.

Figure 15 outlines the sequence of fatigue fracture and crack growth in the wire-laser DED deposits. Rotating bending produces the largest cyclic stress at, or close to, the specimen surface, where cracks therefore tend to initiate. Localized slip and dislocation accumulation in suitably oriented α colonies or at α/β interfaces can generate stress concentrations and microcracks [43]. Once a microcrack becomes dominant, it traverses the propagation region, where striations, propagation steps, and secondary cracks can develop. Ongoing growth reduces the remaining load-bearing section until a dimpled overload region forms.

The substantially longer life of H175 is consistent with its fine, interwoven α morphology. The stable deposition region contained fine acicular α/α′ and compact basketweave α, producing frequent α/β interfaces and local crystallographic changes along possible crack paths. Consequently, the observed crack path was more tortuous and secondary cracks occurred more frequently. These features indicate repeated local crack deflection or interruption, which increased resistance to fatigue-crack growth.

The H350 structure contained coarse lamellar α, pronounced colonies, and more secondary α, all of which provided relatively direct routes for slip localization and crack advance. Its propagation path generally conformed to the lamellar-colony architecture and showed wider striations with fewer secondary cracks. These observations indicate a more rapid, less interrupted propagation process. The enlarged final-fracture area also supports a shorter stable-growth interval and earlier final failure at the imposed stress.

The fatigue contrast can be viewed as the combined consequence of crack-initiation sensitivity and resistance to propagation. In H175, fine acicular/basketweave α interrupted the continuity of favorable propagation routes and promoted deflection, secondary cracking, and a longer growth process. In H350, coarser lamellar α and developed colonies increased directional microstructural continuity, facilitating crack growth once a surface crack formed. This interpretation is consistent with the measured lives of approximately 3 × 10^7^ cycles for H175 and 1 × 10^5^ cycles for H350.

Fatigue behavior in additively manufactured Ti-6Al-4V can be affected by defects, residual stress, surface condition, prior β grain structure, and α morphology. The examined fracture surfaces showed no obvious initiation feature associated with pores, inclusions, or other macroscopic defects. The two groups also had the same alloy system, specimen geometry, machining procedure, and stress-relief schedule. Effects from submicroscopic defects and residual stress cannot be eliminated completely; the life difference is therefore discussed qualitatively with respect to heat-input-dependent α morphology and the corresponding crack paths.

The combined microstructural and fractographic observations rationalize the large difference in durability at the selected stress level. In H175, the refined interwoven α network promoted changes in crack direction and frequent secondary cracking. In H350, coarse lamellar α and substantial colony development created comparatively uninterrupted paths for fatigue-crack growth.

## 5. Conclusions

Wire-laser DED was used to prepare Ti-6Al-4V at four nominal heat-input levels. The study measured the associated changes in prior β grains, α morphology, and tensile response, then compared the extreme input conditions by 500 MPa rotating-bending fatigue tests and fractography. The principal outcomes are summarized below:

(1) Raising nominal heat input increased prior β grain width from about 642 to 1079 μm and mean α-lath width from 0.65 to 1.32 μm. Over the same range, the intragranular structure progressed from fine acicular/basketweave α to coarser lamellar α with clearer colonies.
(2) Heat input primarily influenced ductility rather than strength. Ultimate tensile and yield strengths remained close to 900 and 840 MPa, respectively, whereas elongation decreased from 14.3% in H175 to 10.3% in H350, a reduction of approximately 28%. All conditions fractured predominantly by a ductile mechanism, but high-heat-input specimens showed reduced necking, shallower dimples, and occasional cleavage facets.
(3) At 500 MPa and R = −1, the two heat-input conditions exhibited markedly different high-cycle fatigue lives. All specimens fractured; H175 survived approximately 3 × 10^7^ cycles, while H350 failed at approximately 1 × 10^5^ cycles.
(4) Fatigue crack growth was closely associated with the retained α morphology. H175 displayed finer striations, more secondary cracks, and a more tortuous route, while H350 displayed wider striations and growth paths that followed developed α colonies more closely. The fine interwoven H175 structure thus offered a plausible microstructural basis for its higher crack-growth resistance and longer fatigue life.

## Figures and Tables

**Table 1:** Chemical compositions of the Ti-6Al-4V substrate and wire (wt.%).

Material	Al	V	Fe	C	N	H	O	Ti
Substrate	6.50	4.20	0.17	0.010	0.010	0.0018	0.18	Bal.
Wire	6.19	4.03	0.09	0.007	0.009	0.0030	0.15	Bal.

**Table 2:** Wire-laser DED parameters for Ti-6Al-4V deposits under different nominal heat inputs.

Sample	Deposition Speed (mm/s)	Nominal Heat Input (J/mm)
H175	8	175
H233	6	233
H290	5	290
H350	4	350

## Data Availability

The original contributions presented in this study are included in the article. Further inquiries can be directed to the corresponding authors.
